# Differences in risk factors for surgical site infection between laparotomy and laparoscopy in gastrointestinal surgery

**DOI:** 10.1371/journal.pone.0274887

**Published:** 2022-09-19

**Authors:** Momoe Utsumi, Terumasa Yamada, Kazuo Yamabe, Yoshiteru Katsura, Nariaki Fukuchi, Hiroki Fukunaga, Masahiro Tanemura, Junzo Shimizu, Yoshinori Kagawa, Shogo Kobayashi, Hidekazu Takahashi, Koji Tanaka, Tsunekazu Mizushima, Hidetoshi Eguchi, Nana Nakayama, Kiyoko Makimoto, Yuichiro Doki

**Affiliations:** 1 Division of Health Sciences, Graduate School of Medicine, Osaka University, Suita City, Osaka, Japan; 2 Department of Gastroenterological Surgery, Higashiosaka City Medical Center, Higashiosaka City, Osaka, Japan; 3 Department of Surgery, Kinan Hospital, Tnabe City, Osaka, Japan; 4 Department of Surgery, Kansai Rosai Hospital, Amagasaki City, Hyogo, Japan; 5 Department of Surgery, Suita Municipal Hospital, Suita City, Osaka, Japan; 6 Department of Surgery, Itami City Hospital, Itami City, Hyogo, Japan; 7 Department of Surgery, Rinku General Medical Center, Izumisano City, Osaka, Japan; 8 Department of Surgery, Toyonaka Municipal Hospital, Toyonaka City, Osaka, Japan; 9 Department of Gastroenterological Surgery, Osaka General Medical Center, Osaka City, Osaka, Japan; 10 Department of Gastroenterological Surgery, Graduate School of Medicine, Osaka University, Suita City, Osaka, Japan; 11 Department of Nursing, Kyoto University Hospital, Kyoto City, Kyoto, Japan; 12 Emeritus Professor, Osaka University, Suita City, Osaka, Japan; Hyogo College of Medicine, JAPAN

## Abstract

Extensive gastrointestinal surgery surveillance data in Japan were analyzed to examine the differences in the risk factors for surgical site infection (SSI) between laparotomy and laparoscopic abdominal procedures. Surgical procedures investigated in the study were gastrectomy, cholecystectomy, colectomy, rectal resection, and appendectomy. A total of 32,629 patients were included in the study. The study participants were divided into two groups according to the year of surgery, 2003–2009 (first study period) and 2010–2015 (second study period), due to the increase in the number of laparoscopic surgeries in the second study period. The incidence of SSI was stratified by three SSI classifications (superficial incisional, deep incisional, and organ/space SSI). Multiple logistic regression analysis was performed to predict the risk factors for SSI. The percentage of laparoscopic surgeries performed has increased linearly since 2010. Patients in the second study period were significantly older and had a higher prevalence of SSI risk factors compared with those in the first study period. In addition, the predictive factors changed substantially in most surgical procedures between the two study periods. Wound class ≥ 3 was a ubiquitous risk factor for superficial incisional SSI (SI-SSI) and organ/space SSI (OS-SSI) in both open (laparotomy) and laparoscopic procedures in the first study period. Meanwhile, in the second study period, operative duration was a ubiquitous risk factor in both procedures. The risk factors for SI-SSI differed from those for OS-SSI in the five abdominal surgeries investigated in the study. Periodic examination of risk factors for SSI is recommended in an aging society.

## Introduction

Surgical site infections (SSIs) are the most frequently reported type of healthcare-associated infection, accounting for approximately 20% of all healthcare-associated infections in the United States [1] and in Europe [2] SSIs lead to reoperation, delayed discharge from hospital, reduced quality of life, and increased mortality rates [3–5]. The incidence of SSI in gastrointestinal surgery is significantly higher than that in surgical procedures on other organ sites [6, 7], ranging from 3.7–6.4% in colorectal surgery [8–11] to 3.6–14.2% in gastric surgery [12–14].

Large-cohort colon surgery studies have shown that laparoscopic surgery is associated with a reduced risk of SSI compared with the risk of SSI in open surgery [15–17]. With technological advancements and surgical applications in surgical procedures, the laparoscopic approach has become the gold standard for many surgeries. The number of laparoscopic colectomies performed in Japan increased from 10% in 2003 to 60% in 2013 [17]. Compared with open surgeries, laparoscopic surgeries are likely to offer faster recovery, shorter hospital stays, faster return to normal activities, smaller surgical scars, and better cosmetic appearance [14, 15, 18].

Numerous studies have demonstrated the preventive effects of laparoscopic surgery against SSI. However, these studies on SSI generally treated laparoscopic procedures as a covariate in a multivariate analysis [16, 18, 19]. Furthermore, it is necessary to differentiate SSI types because patients with organ/space SSIs reportedly have worse outcomes than those with superficial incisional SSIs [14]. Nevertheless, patients with superficial incisional SSIs are also adversely affected by the procedure and have a worse quality of life than those without SSIs [20]. Therefore, the differences in risk factors for SSI between open and laparoscopic surgeries, with control for the three SSI wound classifications and the type of surgery, need to be explored [14, 20].

In this study, we examined and compared the incidence of and risk factors for SSI between laparoscopic and open surgeries, with a control for SSI classification, using large-scale SSI surveillance data of gastrointestinal surgeries performed at several medical facilities in Japan.

## Methods

We used data from extensive SSI surveillance of gastrointestinal surgery conducted as part of a medical safety program at several medical institutions in Japan. The collected risk factors for SSI were those for which data had been submitted, as specified in the nosocomial infection control surveillance project of the Ministry of Health [19], Labour, and Welfare. These items are similar to those collected for SSI surveillance by the United States Centers for Disease Control and Prevention [21]. Data on the use of intra-abdominal silk for intraperitoneal suturing were also collected. This is because silk thread is more often used in Japan than in Europe and the United States, where synthetic absorbable thread is more commonly used.

SSI surveillance of gastrointestinal surgery was conducted at two university hospitals and 24 university-affiliated hospitals in the Kansai region of Japan from 2003 to 2015. Data were collected in accordance with the JANIS. The following data were collected from each participating hospital: date of surgery, age, sex, duration of surgery, type of procedure, wound classification, American Society of Anesthesiologists (ASA) score [22], general anesthesia, emergency, endoscopic, and complicated surgery, colostomy, silk suture use, SSI, and SSI classification (superficial incisional, deep incisional, or organ/space SSI). SSI surveillance was conducted as part of a hospital quality improvement program at the participating institutions.

This SSI surveillance data analysis study was approved by the ethics review committee of Osaka University Hospital (approval no. 17380). The surveillance data used in this study were prospectively collected as part of a medical safety program at each healthcare facility. Therefore, written consent was not obtained from the patients in this study. This was clearly stated in the document submitted to the ethics review committee that discussed and approved the study protocol. The dataset used in this study was fully anonymized by the project manager.

### Statistical analysis

The number of laparoscopic procedures performed between 2003 and 2015 was plotted to examine the secular trend of laparoscopic procedures. Subsequently, the average three-yearly SSI rates relating to open and laparoscopic surgery was plotted to assess the fluctuations in the SSI rates. For each year, the mean SSI rate of the past three years was displayed. For example, the SSI rate value for 2005 corresponds to the average SSI infection rate from 2003 to 2005. SSI classification (superficial incisional, deep incisional, and organ/space SSI). Univariate analyses (Student’s t-test and chi-square test) were performed to describe patient characteristics. JMP Basic Pro 15.0.0 software (SAS Institute, Cary, NC, USA) was used for data analysis, and the significance level was set at 5%.

The differences in the prevalence of risk factors between the two study periods were compared after stratification by open and laparoscopic procedures. Univariate logistic regression analysis was performed to screen the variables for multivariate analysis, and those that reached the level of statistical significance were entered into multivariate logistic regression analysis. Patient ages were aggregated into two categories (< 65 and ≥ 65 years). The cutoff point for prolonged surgical time was based on the JANIS Public Information 2015 Annual Report [23]. The following operation duration cutoff points (T-times) were used: 291 min for open gastric surgery and 350 min for laparoscopic gastric surgery; 251 min for open gallbladder surgery and 138 min for laparoscopic gallbladder surgery; 212 min for open colorectal surgery and 264 min for laparoscopic colorectal surgery; 303 min for open rectal surgery and 353 min for laparoscopic rectal surgery; and 88 min for open appendiceal surgery and 91 min for laparoscopic appendiceal surgery [23]. SSI wounds were categorized into semi-clean, contaminated, and suppurated/infected wounds) [24]. The ASA scores were aggregated into two groups (≤ 2 and ≥ 3) [22].

For multiple logistic regression analysis, the differences in risk factors for SI-SSI and OS-SSI between the two study periods were discussed. The prediction of DI-SSI was displayed in Appendix and was not addressed because of the small number of DI-SSI cases in laparoscopic surgery.

## Results

**Fig 1:**
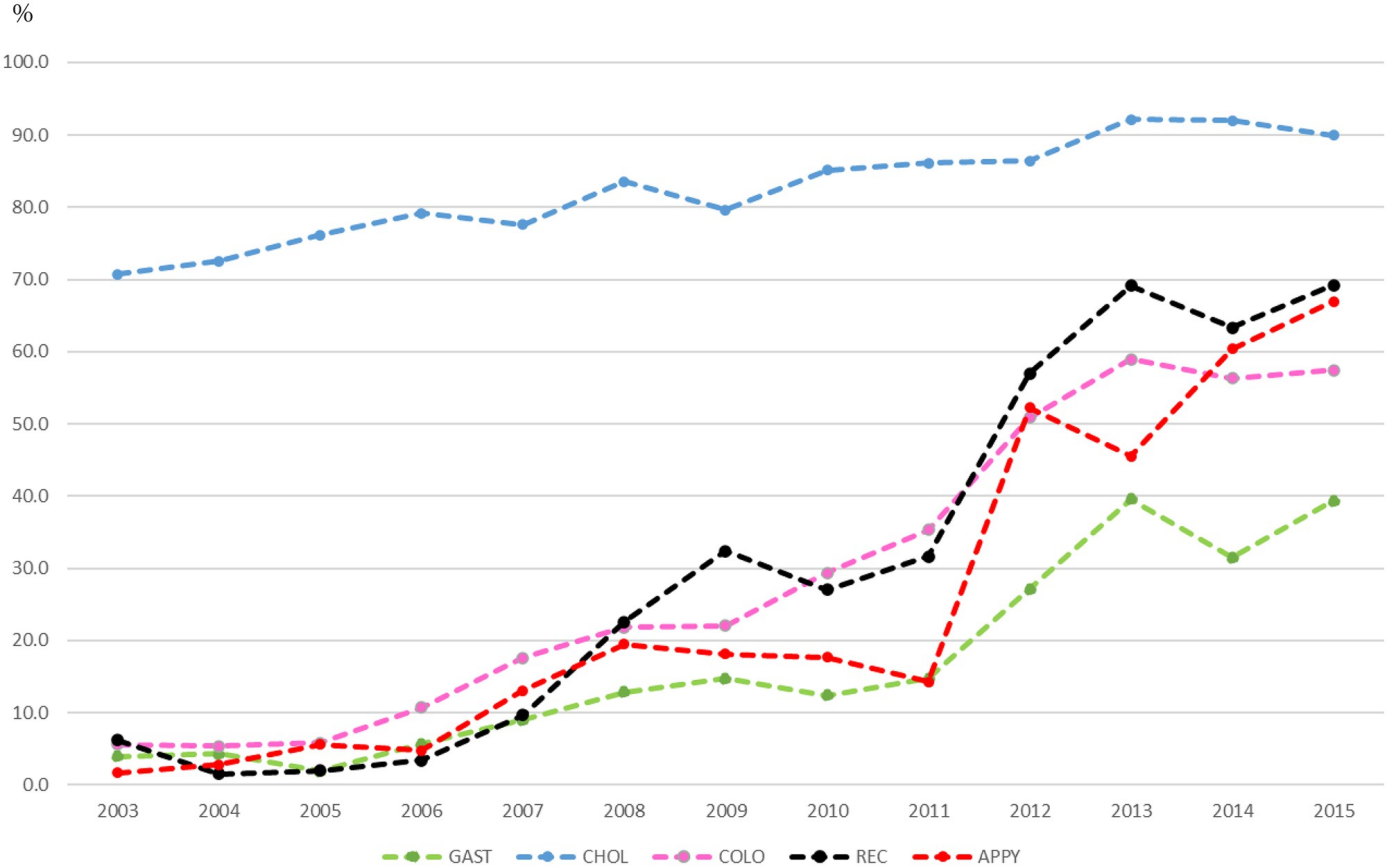
Number of laparoscopic surgeries performed per year (%).

The percentage change in the laparoscopic abdominal surgeries, according to surgical sites, performed between 2003 and 2015 in Kansai region, Japan is shown in Fig 1. In total, 32,629 abdominal surgeries were included in our analysis. Except for cholecystectomy, the proportion of laparoscopic surgery performed in 2003 was < 10%. However, this value increased almost linearly from 2006. By 2015, the proportion of laparoscopic surgeries performed reached 69.2% for rectal surgeries, followed by appendectomy (67.0%), gastric surgery (39.3%), and colectomy (57.5%). Cholecystectomy increased from 70.7% to 90.0% during the study period.

**Fig 2:**
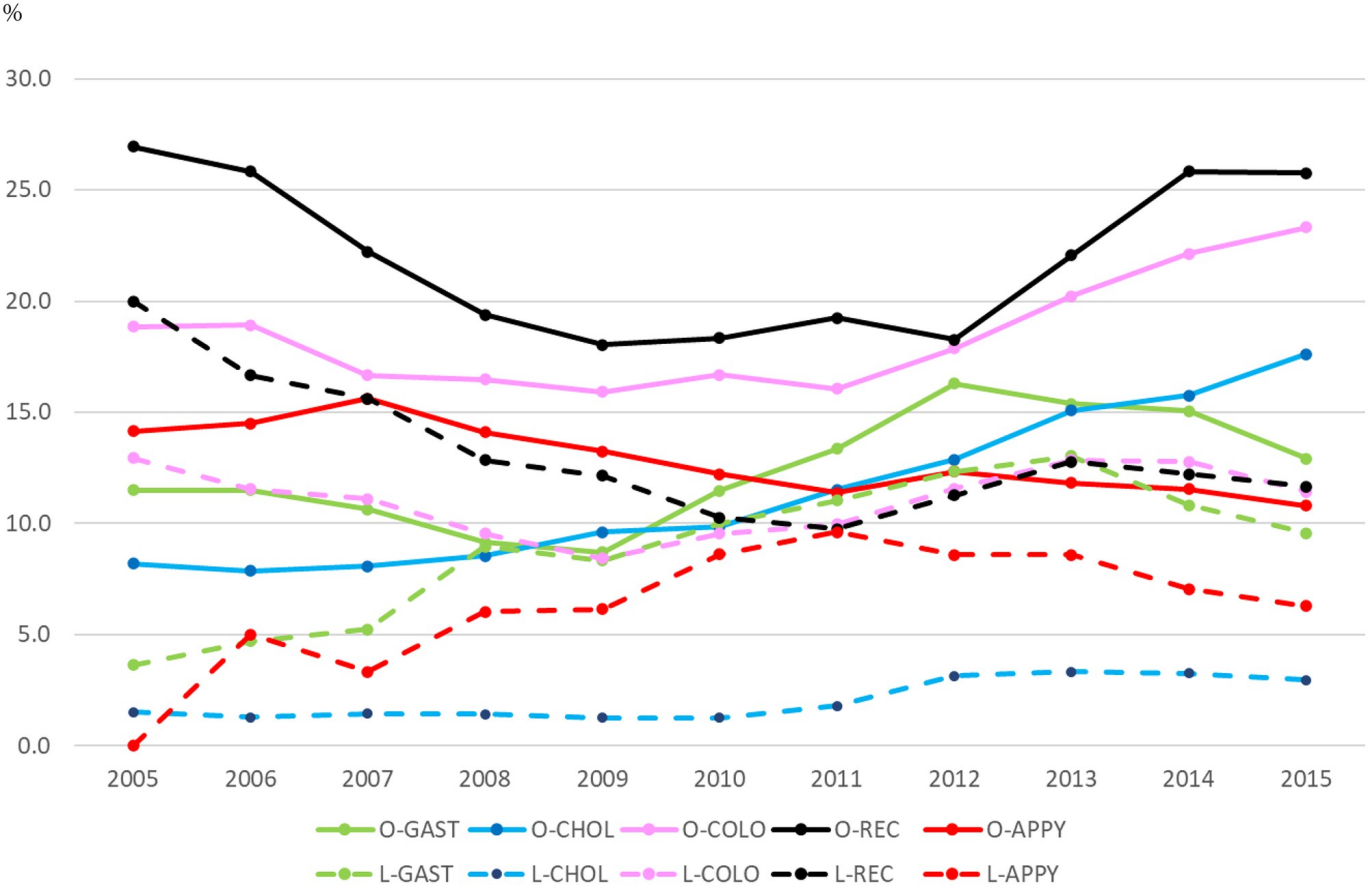
Three-yearly SSI rates average by surgical procedure, 2003–2015.

The three-yearly average of SSI rates by surgical procedures is shown in Fig 2. Except for laparoscopic appendectomy and laparoscopic rectal resection, SSI rates showed a decreasing trend until 2010. Around 2011, the SSI rates started to increase for gastrectomy, colectomy, and cholecystectomy for open procedures, whereas the other procedures showed a decreasing trend. Regardless of the type of surgical procedures, the SSI rates for laparoscopic procedures were higher than those for open procedures in any year.

Table 1 displays the differences in patient demographics and clinical risk factors of SSI between the two study periods according to surgery type. Regardless of the procedure, patients in the second study period were significantly older than those in the first and had a significantly longer duration of surgery. Regarding wound class (clean-contaminated, contaminated, or dirty), the proportion of clean wounds decreased significantly in three open and three laparoscopic procedures. In addition, a significant increase in the proportion of clean wounds was observed in open appendectomy. The proportion of silk sutures dropped in the second study period for all surgery types, except laparoscopic appendectomy, in which the proportion of silk sutures was approximately 1% in both study periods.

**Table 1:** Comparison of demographics and clinical characteristics by year of surgery.

Gastrectomy						
	Open surgery			Laparoscopic surgery		
	2003–2009	2010–2015	p	2003–2009	2010–2015	p
	(n = 5536)	(n = 2195)		(n = 423)	(n = 910)	
Age, years	66.39±11.25	69.87±11.18	<0.0001	62.67±11.42	67.35±12.25	<0.0001
Men	68.05	70.43	0.0414	63.59	66.26	0.3537
Operation duration, min	214.4±73.95	239.69±93.43	<0.0001	225.83±90.75	271.04±101.71	<0.0001
Wound class:			<0.0001			0.0042
clean-contaminated	95.6	93.1		97.6	93.3	
contaminated	2.2	4.3		1.2	4.0	
dirty	2.2	2.6		1.2	2.7	
ASA classification ≥3	9.34	15.81	<0.0001	3.78	10.55	<0.0001
General anesthesia	100	100	ー	100	100	ー
Emergency operation	7.88	5.88	0.0022	3.78	5.93	0.1138
Combined surgery	21.48	6.61	<0.0001	4.49	2.75	0.1017
Stoma	0.29	0.55	0.0954	0.47	4.73	<0.0001
Silk suture	39.41	8.43	<0.0001	18.44	2.75	<0.0001
SSI incidence	10.37	14.08	<0.0001	7.8	9.78	0.2628
Superficial incisional SSI	4.12	7.88	<0.0001	3.07	4.29	0.3619
Deep incisional SSI	0.63	0.82	0.2846	1.42	0.88	0.3994
Organ/space SSI	5.62	5.38	0.7	3.31	4.62	0.3065
Cholecystectomy						
	Open surgery			Laparoscopic surgery		
	2003–2009	2010–2015	p	2003–2009	2010–2015	p
	(n = 925)	(n = 307)		(n = 3140)	(n = 2502)	
Age, years	64.99±12.88	68.14±12.71	0.0002	58.15±13.69	61.61±14.23	<0.0001
Men	59.57	63.84	0.2003	45.92	51.32	<0.0001
Operation duration, min	129.77±67.58	159.71±94.40	<0.0001	94.55±45.51	111.63±57.00	<0.0001
Wound class:			0.3285			<0.0001
clean-contaminated	77.3	79.8		90.5	87.5	
contaminated	14.6	14.7		7.9	11.8	
dirty	8.1	5.5		1.6	0.6	
ASA classification ≥3	13.3	17.92	0.0494	3.44	7.19	<0.0001
General anesthesia	100	100	ー	100	100	ー
Emergency operation	18.92	12.05	0.0052	15.35	8.83	<0.0001
Combined surgery	6.38	1.3	0.0001	0.99	0.2	0.0001
Stoma	0.76	0.65	1	0.1	0.16	0.707
Silk suture	45.95	14.98	<0.0001	1.72	0.92	0.0108
SSI incidence	8.43	14.98	0.0014	1.34	3	<0.0001
Superficial incisional SSI	5.41	11.07	0.0015	1.08	2.6	<0.0001
Deep incisional SSI	0.97	1.3	0.7468	0.06	0.08	1
Organ/space SSI	2.05	2.61	0.6524	0.19	0.32	0.4219
Colectomy						
	Open surgery			Laparoscopic surgery		
	2003–2009	2010–2015	p	2003–2009	2010–2015	p
	(n = 4385)	(n = 2281)		(n = 649)	(n = 2337)	
Age, years	67.74±11.83	69.90±12.87	<0.0001	65.99±11.19	69.76±11.43	<0.0001
Men	54.8	55.33	0.6972	55.47	55.71	0.9289
Operation duration, min	161.56±67.85	183.43±95.85	<0.0001	197.85±69.95	217.32±84.02	<0.0001
Wound class:			<0.0001			0.0430
clean-contaminated	88.7	77.2		97.8	95.8	
contaminated	5.2	11.7		1.9	3.2	
dirty	6.1	11.1		0.3	1.0	
ASA classification ≥3	14.39	25.73	<0.0001	5.7	11.68	<0.0001
General anesthesia	100	100	ー	100	100	ー
Emergency operation	17.65	25.87	<0.0001	4.93	3.38	0.0777
Combined surgery	9.56	3.29	<0.0001	3.85	0.39	<0.0001
Stoma	11.56	22.67	<0.0001	1.39	8.69	<0.0001
Silk suture	40.94	10.57	<0.0001	46.07	4.24	<0.0001
SSI incidence	17.54	20.78	0.0015	9.71	11.68	0.182
Superficial incisional SSI	11.36	15.48	<0.0001	5.55	8.81	0.0071
Deep incisional SSI	2.74	1.71	0.0087	1.39	0.94	0.379
Organ/space SSI	3.44	3.59	0.5706	2.77	1.93	0.2221
Rectal resection						
	Open surgery			Laparoscopic surgery		
	2003–2009	2010–2015	p	2003–2009	2010–2015	p
	(n = 2212)	(n = 762)		(n = 249)	(n = 1021)	
Age, years	65.26±11.17	68.55±11.20	<0.0001	64.64±11.98	67.68±10.58	<0.0001
Men	62.48	61.94	0.7949	62.25	60.72	0.7172
Operation duration, min	229.05±117.58	253.96±134.29	<0.0001	256.61±94.93	296.34±126.91	<0.0001
Wound class:			0.0028			0.5067
clean-contaminated	91.1	88.6		95.6	96.9	
contaminated	6.4	6.3		4.0	2.6	
dirty	2.6	5.1		0.4	0.5	
ASA classification ≥3	9.31	12.86	0.0067	5.22	10.28	0.0143
General anesthesia	100	100	ー	100	100	ー
Emergency operation	7.96	7.35	0.6387	2.01	2.06	1
Combined surgery	9.67	5.64	0.0006	2.41	0.49	0.0102
Stoma	31.92	38.85	0.0005	15.26	24.78	0.0013
Silk suture	47.47	16.14	<0.0001	46.18	7.74	<0.0001
SSI incidence	22.69	22.44	0.9201	14.06	11.56	0.2785
Superficial incisional SSI	11.21	14.96	0.0083	4.42	6.76	0.1925
Deep incisional SSI	2.92	1.18	0.0065	1.61	1.37	0.7658
Organ/space SSI	8.54	6.3	0.0707	8.03	3.43	0.0049
Appendectomy						
	Open surgery			Laparoscopic surgery		
	2003–2009	2010–2015	p	2003–2009	2010–2015	p
	(n = 1325)	(n = 683)		(n = 129)	(n = 658)	
Age, years	46.79±18.41	48.53±18.28	0.045	35.40±14.54	45.38±18.33	<0.0001
Men	57.81	56.22	0.5053	25.58	49.7	<0.0001
Operation duration, min	67.26±34.68	73.81±45.12	0.0003	71.72±35.51	85.93±42.39	0.0004
Wound class:			<0.0001			0.1501
clean-contaminated	47.3	64.0		56.6	48.8	
contaminated	22.2	18.6		27.1	27.8	
dirty	30.5	17.4		16.3	23.4	
ASA classification ≥3	5.89	5.56	0.8402	2.33	7.45	0.0319
General anesthesia	62.19	89.02	<0.0001	99.22	100	0.1639
Emergency operation	94.26	82.72	<0.0001	89.92	69.45	<0.0001
Combined surgery	1.13	0.73	0.4823	2.33	0	0.0043
Stoma	0.15	0.73	0.049	0.78	0.15	0.3011
Silk suture	30.49	7.76	<0.0001	0.78	1.06	1
SSI incidence	14.04	11.13	0.0692	6.2	6.38	1
Superficial incisional SSI	8.3	7.76	0.7303	3.88	5.32	0.6615
Deep incisional SSI	3.09	1.32	0.0152	0.78	0.15	0.3011
Organ/space SSI	2.64	2.05	0.4497	1.55	0.91	0.6241

The SSI rates in open procedures were significantly higher in the second study period than in the first study period for gastrectomy, cholecystectomy, and colectomy. For laparoscopic procedures, the significant increase in the SSI rate was limited to cholecystectomy. After stratification by SSI classification, the SI-SSI rates increased significantly in gastrectomy, colectomy, and rectal resection for open procedures (Table 1), and for laparoscopic procedures, the SI-SSI rates increased in cholecystectomy and colectomy. The DI-SSI rate decreased significantly in rectal resection, appendectomy, and colectomy for open procedures. A significant decrease in DI-SSI was observed in open appendectomy, and there were no other significant changes in SSI rates.

The predictors of SI-SSI and OS-SSI on multiple logistic regression by surgical procedure and study period are shown in Table 2. Notable changes in the risk factors for SI-SSI between the two study periods were observed in the open and laparoscopic gastrectomy groups. Wound class ≥ 3 was the only risk factor in the first study period for these procedures. Meanwhile, there were four risk factors (longer operative duration, ASA classification ≥3, emergency operation, and silk suture) in the second study period l. The number of demographic and clinical risk factors for OS-SSI increased from five in the first study period to six in the second study period for open and laparoscopic gastrectomies.

**Table 2:** Multiple logistic regression analysis of SSI risk factors by surgical procedure: SI-SSI and OS-SSI.

Gastrectomy								
Variables	Open surgery				Laparoscopic surgery			
	SI-SSI		OS-SSI		SI-SSI		OS-SSI	
	2003–2009	2010–2015	2003–2009	2010–2015	2003–2009	2010–2015	2003–2009	2010–2015
Sex (female as a reference)			1.60 (1.21–2.12)	1.76 (1.16–2.67)			1.80 (1.36–2.37)	1.93 (1.27–2.91)
Age: ≥ 65 years			1.66 (1.29–2.13)	1.52 (1.02–2.25)			1.69 (1.32–2.17)	1.53 (1.03–2.26)
Operation duration: ≥ T-time		1.52 (1.12–2.07)	4.24 (3.27–5.49)	2.76 (1.97–3.88)		1.49 (1.00–2.21)	3.51 (2.44–5.04)	2.03 (1.36–3.04)
Wound class: ≥ 3	3.44 (2.12–5.57)		2.32 (1.43–3.79)		3.44 (2.12–5.59)		2.36 (1.45–3.82)	
ASA classification: ≥ 3		1.97 (1.40–2.78)				1.96 (1.39–2.77)		
Emergency operation: Yes		2.77 (1.55–4.98)		2.64 (1.26–5.52)		2.73 (1.52–4.88)		2.40 (1.16–4.96)
Combined surgery: Yes			1.53 (1.19–1.99)	2.67 (1.60–4.47)			1.90 (1.48–2.44)	2.71 (1.61–4.55)
Silk suture: Yes		1.97 (1.20–3.24)		1.98 (1.15–3.41)		1.91 (1.16–3.14)		
Cholecystectomy								
Variables	Open surgery				Laparoscopic surgery			
	SI-SSI		OS-SSI		SI-SSI		OS-SSI	
	2003–2009	2010–2015	2003–2009	2010–2015	2003–2009	2010–2015	2003–2009	2010–2015
Sex (female as a reference)			3.90(1.31–11.56)	5.53 (1.24–24.66)			3.58(1.20–10.64)	
Age: ≥ 65 years	2.43 (1.51–3.91)				2.40 (1.49–3.86)			
Operation duration: ≥ T-time		3.91 (2.21–6.90)	9.05 (3.59–22.86)	5.73 (1.96–16.76)	1.66 (1.02–2.70)	2.93 (1.87–4.59)	5.46 (2.24–13.31)	15.02 (3.31–68.20)
Wound class: ≥ 3	2.29 (1.36–3.85)	1.80 (1.04–3.11)	4.39 (1.89–10.18)		2.16 (1.28–3.64)		4.22 (1.83–9.72)	
ASA classification: ≥ 3	2.21 (1.19–4.12)				2.18 (1.17–4.05)			
Emergency operation: Yes	0.42 (0.21–0.84)							
Stoma: Yes						9.84 (1.09–88.80)		
Silk suture: Yes	3.00 (1.84–4.89)	27.70 (15.79–48.59)	2.40 (1.01–5.70)		2.74 (1.67–4.49)	25.68 (14.61–45.14)		
Colectomy								
Variables	Open surgery				Laparoscopic surgery			
	SI-SSI		OS-SSI		SI-SSI		OS-SSI	
	2003–2009	2010–2015	2003–2009	2010–2015	2003–2009	2010–2015	2003–2009	2010–2015
Sex (female as a reference)				1.52 (1.03–2.22)				1.53 (1.05–2.24)
Age: ≥ 65 years			0.72 (0.52–1.00)					
Operation duration: ≥ T-time			2.28 (1.62–2.21)	2.81 (1.94–4.07)			2.94 (1.93–4.50)	2.89 (1.96–4.26)
Wound class: ≥ 3	2.51 (1.90–3.33)	1.97 (1.52–2.55)	2.09 (1.37–3.20)		2.54 (1.92–3.37)	1.98 (1.53–2.57)	2.19 (1.43–3.35)	
ASA classification: ≥ 3			1.53 (1.02–2.30)	1.63 (1.07–2.49)			1.52 (1.01–2.29)	1.62 (1.07–2.47)
Emergency operation: Yes		1.45 (1.12–1.88)	2.27 (1.54–3.35)			1.44 (1.12–1.87)	2.30 (1.56–3.39)	
Combined surgery: Yes		0.22 (0.08–0.63)	1.69 (1.10–2.61)			0.22 (0.08–0.62)	1.65 (1.06–2.57)	
Stoma: Yes	1.77 (1.35–2.31)	1.33 (1.04–1.69)		2.66 (1.74–4.05)	1.77 (1.35–2.32)	1.31 (1.03–1.66)		2.45 (1.61–3.72)
Silk suture: Yes		3.32 (2.54–4.33)		2.45 (1.44–4.16)		3.30 (2.53–4.31)		2.42 (1.43–4.09)
Rectal resection								
Variables	Open surgery				Laparoscopic surgery			
	SI-SSI		OS-SSI		SI-SSI		OS-SSI	
	2003–2009	2010–2015	2003–2009	2010–2015	2003–2009	2010–2015	2003–2009	2010–2015
Sex (female as a reference)			2.58 (1.81–3.69)				2.61 (1.83–3.73)	
Operation duration: ≥ T-time		1.52 (1.09–2.14)	1.70 (1.19–2.43)			1.50 (1.03–2.17)		
Wound class: ≥ 3	1.92 (1.28–2.86)		2.74 (1.80–4.18)	2.23 (1.08–4.61)	1.92 (1.28–2.87)		2.70 (1.77–4.11)	2.23 (1.08–4.61)
Emergency operation: Yes		2.50 (1.34–4.67)				2.42 (1.30–4.50)		
Stoma: Yes	2.65 (1.99–3.52)	1.60 (1.15–2.23)			2.64 (1.99–3.50)	1.59 (1.13–2.22)		
Silk suture: Yes		2.19 (1.41–3.38)				2.14 (1.39–3.30)		
Appendectomy								
Variables	Open surgery				Laparoscopic surgery			
	SI-SSI		OS-SSI		SI-SSI		OS-SSI	
	2003–2009	2010–2015	2003–2009	2010–2015	2003–2009	2010–2015	2003–2009	2010–2015
Operation duration: ≥ T-time		1.82 (1.13–2.92)		5.30 (1.93–14.58)		1.79 (1.11–2.88)	2.18 (1.07–4.45)	4.69 (1.78–12.41)
Wound class: ≥ 3	2.65 (1.64–4.28)		14.26 (3.36–60.55)		2.64 (1.63–4.27)		14.10 (3.32–59.92)	
ASA classification: ≥ 3	1.88 (1.00–3.51)	2.46 (1.24–4.87)			1.89 (1.01–3.54)	2.43 (1.22–4.82)		
Stoma: Yes				96.03(10.81-852-75)				89.97(10.31–785.29)
Silk suture: Yes	1.57 (1.04–2.37)	10.31 (5.57–19.10)		5.87 (1.70–20.22)	1.58 (1.05–2.39)	10.44(5.64–19.36)		6.05 (1.76–20.80)

Furthermore, an increase in the number of risk factors for SI-SSI between the study periods was observed for open and laparoscopic colectomies and rectal resections. The wound class ≥ 3 and stoma were the only risk factors of SI-SSI for these four procedures. In the second study period, emergency operation, silk suture, and the other well-known risk factors were retained, in addition to the wound class and stoma. In rectal resection, the number of risk factors for OS-SSI decreased in open and laparoscopic procedures, and a wound class ≥ 3 was the only risk factor for OS-SSI in the second study period.

For colectomy, classic risk factors for OS-SSI, such as duration of surgery, wound class, and ASA score were significant predictors in the first study period for both open and laparoscopic procedures. However, in the second study period, operative duration and ASA score were the major risk factors between the two study periods for both open and laparoscopic procedures. Male sex, stoma, and silk sutures were additional risk factors in the second study period.

For both open and laparoscopic appendectomy, the number of risk factors for SI-SSI did not change between the two study periods, and the ASA score and silk suture were the common risk factors between the two study periods. For OS-SSI, wound class was retained as a risk factor in open appendectomy in the first study period. However, in the second study period, three different risk factors emerged, including operative duration and silk suture. For OI-SSI in a laparoscopic procedure, operative duration was the common risk factor between the two study periods, and wound class was only a significant predictor in the first study period.

The number and composition of risk factors for SSI changed substantially from the first to the second study period for both SI-SS and OS-SSI as well as in open and laparoscopic procedures. In the first study period, wound class ≥ 3 was a ubiquitous risk factor for SI-SSI and OS-SSI in both open and laparoscopic procedures. Meanwhile, in the second study period, operative duration was a ubiquitous risk factor in these procedures. The ASA score was less prevalent than the wound class or operative duration as a risk factor and was likely to be retained in the model with either the wound class or operative duration in both study periods. Within the SSI classification category, the risk factors for SSI in open procedures were similar to those in laparoscopic procedures, although there were some differences. The risk factors for SI-SSI differed from those for OS-SSI in the five abdominal surgeries considered in our study.

## Discussion

Our large abdominal surgery cohort revealed an increasing trend in the number of laparoscopic surgeries being performed. In addition, the prevalence of SSI risk factors significantly increased during the second study period. The SI-SSI rates increased in four open and two laparoscopic gastrointestinal procedures. The adjusted SSI risk factors changed substantially between the two study periods after categorization by surgical procedure, laparoscopic status, and SSI wound classification.

The increase in the proportion of laparoscopic procedures reflects the expansion of the indications for laparoscopic procedures in abdominal surgeries during the study period. The similar trend of open surgeries’ replacement with the minimally invasive alternatives during the 16-year period is reported, although the rate of increase in the laparoscopic procedures varies among the six procedures [25]. Laparoscopic procedures cause significantly less tissue damage and more effectively preserve immune system function compared with open procedures, thereby contributing to a lower incidence of infectious complications [26]. A recent meta-analysis of 16 randomized controlled trials of laparoscopic colorectal procedures confirmed this position and showed that laparoscopic procedures, compared with open procedures, significantly lower the risk of SSI [18].

The decreases in SSI rates for most procedures in the 1^st^ half of the study period may reflect the Hawthorn effect of the surveillance. A systematic review by Abbas reported similar findings. The review showed that SSI rates were lower than at baseline during the first five years of joining the surveillance network; however, they subsequently returned to the baseline level [27]. In our study, the SSI rates for laparoscopic appendectomy and laparoscopic gastrectomy increased in the 1^st^ study period, which cannot be explained by the Hawthorn effect.

The increased SSI rates in the 2^nd^ study period may reflect the higher proportion of patients with major SSI risk factors in the 2^nd^ study period, i.e., longer operative duration, the increased proportion of ASA score ≧ 3 and wound class contaminated/dirty, and older age. Despite the increased prevalence of multiple risk factors, the SSI rates for laparoscopic procedures show a decreasing trend toward the end of the 2^nd^ study period.

Higher technical skills of the surgeons were associated with lower adverse outcomes, such as unplanned readmission and reoperation in the video assessment study [28]. In Stulberg et al.’s study, no associations were found between surgeons’ technical skills and SSI rates, possibly because no statistical adjustments for SSI risk factors were implemented. In our study, there was no training program for laparoscopic surgery. The increased frequency of laparoscopic procedures may have improved the surgeons’ skills.

The difference in the incidence rate of SSIs between open and laparoscopic procedures was highest for cholecystectomy, and the difference in the proportion of women between these two procedures was also the highest. Endoscopic surgery is generally performed more in women than in men [24]. Women tend to opt for laparoscopy instead of laparotomy because laparoscopy results in less visible surgical wounds. Men who undergo laparoscopic cholecystectomy have a higher risk for conversion to open surgery [26, 29] and intraoperative and postoperative local complications [30] than women. In a large study of patients who underwent gallbladder surgery [31], more men than women had severe biliary diseases (such as acute cholecystitis, obstruction, and bile duct stones) and underwent open surgery. Nevertheless, the increase in the number of men undergoing laparoscopic procedures is limited to appendectomy and cholecystectomy. The proportion of men is expected to increase with these procedures.

In our study, men had an elevated risk of developing OS-SSI. Previous studies have revealed differences in the risk factors for SSI among the three SSI categories in gastrointestinal surgery, showing that male sex is only a risk factor for organ/space SSI and not for superficial incisional SSI [13, 32, 33]. In Japan, men are more likely to have multiple risk factors for SSI compared with women. Although we have no data on these variables, Japan Health and Nutritional Survey show that smoking prevalence was 27.1% among men and 7.6% among women [31]. Additionally, the prevalence of diabetes mellitus based on glycated hemoglobin concentration was twice as high among men than among women (19.7% and 10.8%, respectively) [31]. Furthermore, the prevalence of metabolic syndrome was three times higher among men in their sixties than among women of similar age (33.6% and 12.3%, respectively). Although patient sex cannot be altered prior to surgery, the information is important for risk communication.

Fatigue among physicians and surgical team members reportedly contributes to SSIs [34]. However, operative duration ≥ the T time was a risk factor for OS-SSI in the first study period and both SI-SSI and OS-SSI in the second study period, with varying mean operative durations. In particular, the mean operative duration for appendectomy was one-third that for gastrectomy. It is unlikely that fatigue among surgical team members increases the risk of SSI in common abdominal surgeries.

Operative duration ≥ the T time is considered to reflect the surgeon’s skills and/or complexity of the surgery [34]. For example, gastrectomy is the most common gastrointestinal surgical procedure in Japan because of the high incidence of gastric cancer [35]. In a study comparing the incidence of SSI among three types of gastric surgeries (total gastrectomy, distal gastrectomy, and another type of gastrectomy), the relative risk ratio for total gastrectomy was 1.77 (95% confidence interval, 1.65–1.91) using distal gastrectomy as a reference [36]. Furthermore, a longer operative time involves more manipulation of organs, leading to tissue desiccation and an increase in the risk for bacterial contamination [34]. In our study, preoperative antibiotics were administered to the patients according to established protocols, and additional antibiotics were administered when requested by the physician. We did not have information on the type, dosage, or duration of antibiotic administration.

In our study, we did not have access to clinical information such as comorbidities. An ASA score of ≥ 3 is a surrogate measure of comorbidity. When an ASA score of ≥ 3 was a significant independent predictor of SI-SSI or OS-SSI in our study, a wound class of ≥ 3 was retained in the model. In some studies, the ASA score was a predictor of overall SSI [37, 38]. In a United States large colon surgery study, ASA score ≥ 2 and wound class ≥ 2 were significant predictors of SI-SSI, DI-SSI, and OS-SSI along with other multiple clinical factors [32], whereas laparoscopy was a strong protector against these three classifications of SSIs. As a large clinical database becomes available, it is necessary to develop a better predictor of SSI using clinical comorbidity information.

Older age was a risk factor for OS-SSI in open and laparoscopic gastrectomy in both study periods and a risk factor for SI-SSI in open and laparoscopic cholecystectomies in the first study period. Age was reportedly a risk factor for overall SSIs in univariate analysis but not in multivariate analysis, with laparoscopy serving as a protective factor in abdominal surgery [31, 39]. Since laparoscopy protects against SSIs, age is unlikely to be retained in the final multivariate model with a laparoscopic procedure as a covariate. In our study, when older age was a significant predictor of SSI, wound class ≥3, longer operative duration, and ASA score ≥3 were likely significant. In other words, in the absence of these classical SSI risk factors, age was not retained in the final logistic regression model.

In addition, the use of silk sutures emerged as a risk factor for SSI in open and laparoscopic gastrointestinal surgeries in the second study period, while the use of silk sutures plummeted. Recent studies on suture materials have focused on the efficacy of anti-bacterial coated sutures in preventing SSI. A meta-analysis of the effectiveness of triclosan-coated sutures based on 25 randomized controlled trials involving various surgical procedures showed that the use of such sutures significantly reduced the risk of SSI compared with standard sutures in clean and contaminated surgery [40]. Triclosan-coated sutures were not significantly effective for the prevention of superficial or deep incisional SSI and may not reduce the incidence of SSI. However, the consequences of organ/space SSI are substantial in terms of cost of care and reduced quality of life. Further research on antibacterial sutures is required.

In this study, the SSI surveillance team followed up the patients for 30 days. Nevertheless, SSIs were likely to be diagnosed after patients are discharged because of their short hospital stay. Reports from surgeons and patients have indicated that some SSIs manifest several months postoperatively [41, 42]. Patient education regarding potential SSIs and the enforcement of surveillance systems are, therefore, essential.

## Limitations

The study included many gastrointestinal surgical procedures, a long SSI observation period, and the participation of multiple institutions. Following standard SSI surveillance methods, accurate data were prospectively collected with minimal under-reporting. However, the study has some limitations. A limited number of variables were collected and analyzed. The indications for and complexity of surgery were not considered in our analysis. Bowel obstruction, perforation, and complex inflammation are difficult to treat and require advanced laparoscopic techniques. Additionally, we did not identify patients’ previous surgeries or the extent of adhesions, which are well-known factors that influence the planning of surgical approaches. We were unable to examine several other clinical variables, such as smoking history and blood transfusion, which are also considered risk factors for SSI. However, it is unlikely that these factors affected only one group of patients who underwent either open or laparoscopic surgery.

We excluded DI-SSI from the multivariate analysis because of the small number of infections in laparoscopic procedures. However, the risk factors for DI-SSI are different from those for OS-SSI, as shown in the appendix, and these two SSI classifications were not aggregated.

## Conclusion

In this study, we examined the risk factors for SSI in patients undergoing open and laparoscopic gastrointestinal surgeries, according to SSI type (superficial incisional, deep incisional, or organ/space SSI). An increasing number of patients at high risk of SSI seems to have resulted in an increased incidence of SI-SSI. Separate multivariate analyses of laparoscopy and laparotomy showed different risk factors for SSI; thus, we recommend that laparoscopy and laparotomy be analyzed separately rather than as covariates.
